# Cold Atmospheric Plasma Reduces Vessel Density and Increases Vascular Permeability and Apoptotic Cell Death in Solid Tumors

**DOI:** 10.3390/cancers14102432

**Published:** 2022-05-14

**Authors:** Philipp Kugler, Sven Becker, Christian Welz, Nadine Wiesmann, Jonas Sax, Christoph R. Buhr, Markus H. Thoma, Juergen Brieger, Jonas Eckrich

**Affiliations:** 1 Department of Otorhinolaryngology, University Medical Center Mainz, 55131 Mainz, Germany; pkugler@students.uni-mainz.de (P.K.); nwiesman@uni-mainz.de (N.W.); r.ch.buhr@gmail.com (C.R.B.); brieger@uni-mainz.de (J.B.); 2 Department of Otorhinolaryngology, Head and Neck Surgery, University of Tübingen Medical Center, 72016 Tübingen, Germany; sven.becker@med.uni-tuebingen.de; 3 Department of Otorhinolaryngology, Head and Neck Surgery, University Medical Center Göttingen, 37075 Göttingen, Germany; kontakt@hno-muc-nord.de; 4 Department of Oral and Maxillofacial Surgery—Plastic Surgery, University Medical Center Mainz, 55131 Mainz, Germany; jonsax@students.uni-mainz.de; 5 Institute of Experimental Physics I, Justus Liebig University Giessen, 35392 Giessen, Germany; markus.h.thoma@exp1.physik.uni-giessen.de; 6 Department of Otorhinolaryngology, University Medical Center Bonn (UKB), 53127 Bonn, Germany

**Keywords:** plasma medicine, tumor vasculature, experimental tumor research, in vivo experimentation, CAM assay

## Abstract

**Simple Summary:**

Cold atmospheric plasma (CAP) resembles a physical state of matter, best described as ionized gas. CAP has demonstrated promising anti-cancer effects. Despite their relevance for the treatment of solid tumors, effects of CAP on tumor vessels and tumor-blood-circulation are still insufficiently investigated. CAP exposure reduced the vessel network inside the tumor and increased vascular leakiness, leading to an elevated tumor cell death and bleeding into the tumor tissue. These effects highlight the potential of CAP as a promising and yet underrated therapeutic modality for addressing the tumor vasculature in the treatment of solid tumors.

**Abstract:**

Cold atmospheric plasma (CAP) has demonstrated promising anti-cancer effects in numerous in vitro and in vivo studies. Despite their relevance for the treatment of solid tumors, effects of CAP on tumor vasculature and microcirculation have only rarely been investigated. Here, we report the reduction of vessel density and an increase in vascular permeability and tumor cell apoptosis after CAP application. Solid tumors in the chorioallantoic membrane of chicken embryos were treated with CAP and evaluated with respect to effects of CAP on embryo survival, tumor size, and tumor morphology. Furthermore, intratumoral blood vessel density, apoptotic cell death and the tumor-associated microcirculation were investigated and compared to sham treatment. Treatment with CAP significantly reduced intratumoral vessel density while increasing the rate of intratumoral apoptosis in solid tumors. Furthermore, CAP treatment increased vascular permeability and attenuated the microcirculation by causing vessel occlusions in the tumor-associated vasculature. These effects point out the potential of CAP as a promising and yet underrated therapeutic modality for addressing the tumor vasculature in the treatment of solid tumors.

## 1. Introduction

Plasma describes a physical state of matter resembling ionized gas. It is naturally present in the ionosphere or during lightning strikes. Since the 1970s, plasma has been used in medicine, mainly for indications like instrument sterilization and tissue cauterization [1]. The more recent development of cold atmospheric plasma (CAP) diversified the applicability of plasma in clinical practice by improving the applicational safety and feasibility during clinical use. CAP is often described as weakly ionized gas and can be locally produced in ambient air or noble gases [2]. Utilization of plasma at low temperature and atmospheric pressure enables the direct application on living cells and tissues without major impairment or destruction [3]. CAP contains high levels of charged particles, radicals, electromagnetic radiation, and electric fields [4,5]. These components interact with surrounding gases and liquids by producing a mixture of reactive oxygen and nitrogen species (RONS), such as HO·, ^1^O_2_, O_2_·−, H_2_O_2_, O_3_, NO and NO_2_ [6,7]. High levels of RONS induce oxidative stress in cells and tissues, thus triggering several intracellular signaling pathways eventually leading to cell death or cell cycle arrest [4,8]. In clinical studies, the use of CAP has mainly been focused on the treatment of chronic wounds and the reduction of bacterial colonization [3]. In recent years, however, scientific interest in CAP as a promising and innovative modality for the treatment of cancer has significantly increased [9,10].

The anti-cancer effects of CAP have been demonstrated in numerous in vitro and in vivo studies [4]. Nonetheless, the underlying mechanisms are still not fully understood. CAP seems to induce several cellular responses including apoptosis, senescence, growth inhibition, and immunogenic cell death [4]. RONS are considered a key component with respect to the CAP–tissue interaction since effects similar to CAP treatment can also be induced by application of RONS-enriched liquids and inhibited by RONS scavengers like N-acetyl-cysteine (NAC) [11,12]. Increasing intracellular concentrations of reactive species may lead to oxidative damage on macromolecules, including DNA and cell membranes. These effects may ultimately result in the activation of mitochondria-mediated apoptosis, in addition to tumor necrosis factor- and p53-mediated apoptotic cell death [2].

There is evidence that CAP treatment might selectively harm cancer cells, which further increased scientific interest in CAP as an innovative tumor therapy. Several theories attribute the selectivity either to the higher metabolic activity of malignant cells [13,14] or to the comparatively increased average density of aquaporins in tumor cells, resulting in an increased permeability of the cell membrane for RONS [15,16,17]. Other authors attributed the effects to the lower concentration of cholesterol in the cell membranes of malignant cells, leading to an increased RONS-derived pore formation [18,19].

In several in vivo studies, CAP treatment resulted in a reduction of tumor volume and tumor growth while increasing the number of apoptotic cells [20,21,22]. Although tumor angiogenesis and a sufficient blood supply have been demonstrated to be a prerequisite for tumor progression and tumor growth, the impact of CAP on tumor vessels and angiogenesis has rarely been investigated thus far. Angiogenesis is induced by pro-angiogenic factors, including VEGF (vascular endothelial growth factor) and PDGF (platelet-derived growth factor) [23,24,25]. Hypoxia in the tumor tissue induces the release of these factors, resulting in an increased neoangiogenesis and an improved tumoral blood supply [23]. More specifically, the angiogenic factors induce migration of endothelial cells, sprouting of new blood vessels in the tumor tissue, and an increased blood vessel permeability [26,27,28]. CAP might interfere with these angiogenic processes, as a reduction of VEGF level was described in two malignant cell lines following CAP treatment [29,30]. Haralambiev et al. recently demonstrated the inhibiting properties of CAP on human dermal microvascular endothelial cells (HDMEC) [26]. Treatment with CAP for 15 s reduced cell growth, metabolic activity, tube formation, and migration while inducing apoptosis in HDMEC in vitro. These findings confirm earlier observations of inhibiting effects of CAP on human aortic endothelial cells (HAEC) [31]. In conclusion, CAP does not only inhibit proliferation and induce apoptosis in malignant cells, but may also affect tumor vasculature and the tumor microenvironment.

To evaluate the impact of CAP on vessel integrity and induction of cell death in solid tumors, the chorioallantoic membrane (CAM) assay was used in this study. The CAM assay has gained increasing popularity as an in vivo tumor model replacing complex and expensive rodent models [32,33] and has already been used to investigate the effects of CAP by other working groups [11,34]. The chicken embryo’s natural immunodeficiency allows xenografts to grow on the non-innervated yet well-vascularized CAM without causing pain or impairment for the animal [35,36]. The unhindered observability of tumors in the CAM enables the in vivo monitoring of tumor growth and vascularization and qualifies the CAM assay as a suitable model for studying tumor angiogenesis [32,37]. In this study, we evaluated the impact of CAP on tumor vessel integrity and mechanisms resulting in tumor impairment. CAP treatment resulted in reduced tumor vessel density while increasing vascular permeability. Furthermore, we detected a relative increase in intratumoral apoptosis after CAP exposure compared to sham treatment.

## 2. Materials and Methods

### 2.1. Cell Culture

The human hepatocellular carcinoma cell line HuH7 [38] were originally purchased from the RIKEB BioResource Center (Kyoto, Japan) and propagated at 37 °C and 5% CO_2_ in Dulbecco’s Modified Eagle Medium (Gibco Life Technologies Limited, Paisley, UK) with 5% (*v*/*v*) fetal calf serum (VWR Seradigm, Radnor, PA, USA) and 2% (*v*/*v*) penicillin/streptomycin (Sigma-Aldrich, St. Louis, MO, USA). HuH7 cells between passage 16 and 26 were used for experimentation, and cells were tested for mycoplasma contamination using the Venor GeM^®^ Classic (Minerva Biolabs^®^, Berlin, Germany) mycoplasma detection kit according to the manufacturer’s instructions. One day before transferring HuH7 cells onto the CAM, three-dimensional (3D) cell cultures were assembled using the basement membrane matrix Matrigel™ (Corning™, Brumath, France) [39]. Cells were harvested by tryptic digestion, counted using a Neubauer counting chamber, and distributed in 1.5 mL tubes with 5 Mio. cells each. After centrifugation for 8 min at 1500 rpm, the cell pellet was mixed with 25 µL ice-cooled Matrigel™ and incubated on a six-well plate for 30 min at 37 °C (Greiner, bio-one International GmbH, Kremsmünster, Austria). After incubation, the Matrigel™ had set to a firm consistency and the 3D cell cultures were subsequently covered with culture medium to incubate overnight.

### 2.2. Chorioallantoic Membrane Assay (CAM Assay)

Although the CAM assay is not considered an animal experiment and experimentation does neither require approval by a governmental organization nor an ethics committee for animal experimentation if the chickens are not hatched, all experiments were performed in accordance with governmental and institutional guidelines for animal experimentation by qualified personnel (Felasa C).

White Leghorn hens’ eggs were cleaned and incubated horizontally at 37.5 °C and 60% relative humidity without rotating the eggs (Brutmaschinen-Janeschitz GmbH, Hammelburg, Deutschland). To lower the level of the developing blastodisc, 6 mL albumen was extracted with a sterilized syringe on day 3 of incubation. The shell was then opened and partially removed with surgical scissors. The opening in the shell was covered with Parafilm^®^ (Bemis Company Inc., Neenah, WI, USA) to reduce evaporation. Cultivation of tumor cells on the CAM started by day 7, when a well-vascularized spot was carefully incised using a single-use scalpel (Feather, Dr. Junghans Medical GmbH, Bad Lausick, Germany) and the 3D culture with 5 Mio. HuH7 cells was placed onto the incision. To avoid desiccation of the tumor cells, 20 µL of Matrigel™ was pipetted onto the culture and the incubation was eventually continued as previously described [35]. Post-therapeutic survival status was assessed daily. Furthermore, thickening of the CAM resembled by opacity of the CAM tissue was documented daily and compared between groups.

### 2.3. CAP Treatment

The CAP device miniFlatPlaSter^®^ was used for tumor treatment. It uses surface micro discharge (SMD) technology to create indirect cold plasma in ambient air [40]. A comprehensive characterization of the emitted components has been published by Welz et al. [10] and Boxhammer et al. [41].

For ultrasonographical and immunohistochemical examination, CAP treatment was performed on four consecutive days (days 10–13 of incubation) with 60 s of continuous CAP exposure per day. Tumor-bearing eggs were allocated to groups (ultrasonography: CAP *n* = 17, Control *n* = 15; immunohistochemistry: CAP *n* = 51, Control *n* = 44) and placed in a sealed container with the CAP device incorporated in the lid (Figure S1). This avoided air circulation and ensured the CAP device was located at an average distance of 5 mm from the CAM bearing the tumor. After CAP or sham treatment for 60 s, eggs were again sealed with Parafilm^®^ and incubation was continued. For sham treatment, eggs were placed inside the homologue air-sealed container for 60 s without Parafilm^®^. After removal from the container, eggs were sealed with Parafilm^®^ before continuation of incubation.

Intravital fluorescence microscopy was performed after the intravascular injection of fluorophore solution into the vascular network. Depending on the specific cohort, tumor-bearing eggs received a single CAP or sham treatment for 60 s immediately before placing the egg under the microscope. Eggs were subsequently placed on a heating panel (MEDAX GmbH & Co KG, Neumünster, Germany) under the microscope and intravital microscopy was performed consecutively for 60 min.

For the quantitation of VEGF-mRNA in the CAM tissue, native eggs without tumors in the CAM were exposed to CAP or sham treatment for four consecutive days (days 10–13 of incubation) with 60 s of CAP or sham exposure, respectively, before excising the CAM on day 14 of incubation for quantitative RT-PCR.

### 2.4. Histological Preparation and Immunohistochemistry 

On day 14 of incubation, 24 h past the last treatment, the embryo was sacrificed by decapitation and the tumor within the surrounding CAM was excised with surgical scissors. It was then transferred into a plastic cassette (Carl Roth GmbH + Co. KG, Karlsruhe, Germany), fixed in formalin solution 4% (*v*/*v*) overnight (VWR International bvba, Leuven, Belgium) and dehydrated in isopropanol solutions with increasing concentrations (80%/90%/100%). After incubation in xylene (AppliChem GmbH, Darmstadt, Germany), each specimen was eventually imbedded in paraffin and cut into 5 µm slides with a microtome (Leica CM1900, Leica Biosystems Nussloch GmbH, Nußloch, Germany).

Hematoxylin-eosin (HE) and immunohistochemical staining for alpha smooth muscle actin (α-SMA) (vessels) and cleaved caspase-3 (apoptosis) were executed according to standard protocols as described previously [35]. For immunohistochemical staining, monoclonal α-SMA antibodies (1/1500, A2547, Sigma-Aldrich, St. Louis, MO, USA) and monoclonal cleaved caspase-3 antibodies (1/50, Asp175, Cell Signaling Technology, Cambridge, UK) were used as first antibodies. For secondary antibodies, biotinylated polyclonal goat anti-mouse immunoglobulin (P 0447, Dako Denmark A/S, Glostrup, Denmark) was added and visualized using horseradish peroxidase-conjugated streptavidin (1/250, Dako Denmark A/S, Glostrup, Denmark).

Stained histological slides were finally digitalized at 100× (α-SMA) and 200× (cleaved caspase-3) magnification using the Nikon Eclipse TE2000 Inverted Microscope with its inbuilt camera system and the NIS-Elements software (Nikon Corp. Chiyoda, Japan). To achieve higher resolution, partial images were fused with the stitching plugin developed by Preibisch et al. [42] for ImageJ version 1.52o (Wayne Rasband, National Institutes of Health, Bethesda, MA, USA).

In each cleaved caspase-3 slide, three randomly placed columns consisting of stacked regions of interest (ROI) of 400 × 100 µm each, were placed within the tumor (Figure 3a,b). Depending on tumor thickness, each column consisted of two to eight ROI per column. Each ROI was evaluated separately for apoptotic cell quantity in relation to the investigated area. The location of each ROI within the column allowed for comparison of apoptotic cell rate in different tumor depths in reference to the tumor surface.

In α-SMA slides, distinct morphological differences of tumor and CAM tissue enabled tumor area measurement in each slide. The mean vessel density was then calculated by analyzing the quantity of stained blood vessels in the tumor tissue and the total area of tumor tissue on the slide using ImageJ.

### 2.5. VEGF Gene Expression Analysis

To evaluate VEGF gene expression in the CAM tissue, mRNA isolation and quantitative real-time polymerase chain reaction (RT-PCR) were performed as described previously [43]. Prior to CAM excision on day 14 of incubation, native eggs without tumors received CAP or sham treatment for four consecutive days (days 10–13 of incubation). CAM tissue was then immediately transferred to cryotubes and stored in liquid nitrogen to avoid RNA degradation. After tissue homogenization, RNA was isolated using the RNeasy Mini Kit (Qiagen N.V., Venlo, The Netherlands), and RNA concentration and purity were evaluated with the NanoDrop™ One (Waltham, MA, USA). Following the RNA to cDNA transcription with iScriptTM cDNA Synthesis Kit 100 (Bio-Rad Laboratories Inc., Hercules, CA, USA), the RT-PCR was performed in duplicate with 5 µL of cDNA in each PCR reaction, using the iTaq Universal SYBR Green Supermix (Bio-Rad Laboratories Inc., Hercules, CA, USA) with the CFX Connect Real-Time PCR Detection System (Bio-Rad Laboratories Inc., Hercules, CA, USA). Data analysis was performed with the Bio-Rad CFX Manager software (Bio-Rad Laboratories Inc., Hercules, CA, USA). For endogenous references, the beta-actin gene and the HRPT1 gene (Hypoxanthine-guanine phosphoribosyltransferase) were used. Quantification of the transcripts was performed according to the ∆∆CT method by comparing the gene expression levels to an internal calibrator sample, thus the displayed expression levels are relative levels. Primers used for RT-PCR are listed in Table 1.

### 2.6. Ultrasonography

On day 14 of incubation, after 240 s of cumulated CAP or sham treatment, *in ovo* ultrasonography was performed to assess tumor volume and vasculature as previously described by our working group [35]. The GE Healthcare Ultrasound LOGIQ E9 (GE Healthcare, Little Chalfont, UK) 15 MHz linear transducer was used in B-Mode (Gain 35) for ultrasonographical imaging. The space between the CAM and shell opening was filled with an average of 4 mL NaCl 0.9% (*w*/*v*) to allow transduction of ultrasound waves. Tumors were visualized in both longitudinal and transversal axes to enable a consistent assessment of the tumor tissue. The respective images were frozen using the “Freeze” function and sagittal, transversal, and coronar tumor diameters were documented. Tumor volume was calculated by using the triaxial ellipsoid formula:

(V = 4/3 × π × (0.5 × d1(sagittal)) × (0.5 × d2(transversal) × 0.5 × d3(coronar)))

Intratumoral hemorrhages, resembled by areas of low echogenicity in the tumor tissue, were evaluated. The NaCl solution was removed after each measurement using an electrical pipette (INTEGRA Biosciences GmbH, Biebertal, Germany) with a 10 mL sterile tube (Greiner CELLSTAR^®^ serological pipette, Greiner AG, Bischofsheim, Germany).

### 2.7. Intravital Fluorescence Microscopy

For intravital examination of tumor-associated blood vessels, tumor-bearing eggs did not receive any treatment until day 14 of incubation. To visualize blood flow and examine vascular permeability, a solution of 70 kDa Tetramethylrhodamine isothiocyanate-Dextran (TRITC-dextran) (TdB Labs AB, Uppsala, Sweden) was prepared according to Egawa et al. [44] by dissolving 500 mg of TRITC-dextran (70 kDa) in 500 µL Dulbecco’s Phosphate Buffered Saline (Sigma-Aldrich, St. Louis, MO, USA). After intravenous injection of 50 µL TRITC-solution, eggs were immediately allocated to the CAP or control group and received 60 s CAP treatment or sham treatment as described above. Eggs were placed horizontally under the intravital microscope (Olympus BXFM, Olympus GmbH, Hamburg, Germany) and a well-vascularized CAM region next to the tumor tissue was selected for intravital fluorescence microscopy using the inbuilt CY3 filter (excitation filter 545 nm, emission filter 605 nm, Chroma Technology Corp, Bellows Falls, VT, USA) and the cellSens Dimension 1.14 software (Olympus GmbH, Hamburg, Germany). The CAM region was visualized once before the treatment and repetitively after the treatment for 1 h (0, 1, 2, 3, 4, 5, 6, 7, 8, 9, 10, 15, 20, 25, 30, 40, 50, and 60 min after treatment). At each timepoint, one image and video sequence were acquired and examined for blood vessel and tissue alterations. Using the Cap-Image 8.10.1 software (Zeintl Software, Heidelberg, Germany) for blood velocity and vessel diameter measurements, segmental blood flow was measured exemplarily after CAP and sham treatment.

### 2.8. Data Management and Statistical Analysis

Statistical analysis was performed using SPSS version 23 (IBM, Armonk, NY, USA). All data sets were analyzed for normal distribution using the Shapiro–Wilk test. As mentioned in the specific sections in detail, comparative tests between two groups were carried out with the student’s *t*-test or the Mann–Whitney U test, depending on whether normal distribution was assumed for the corresponding data sets or not. A *p* < 0.05 was considered significant. Multi-group analysis was carried out using the Kruskal–Wallis test. Survival and CAM-opacity of tumor-bearing eggs was compared between individual groups by performing Kaplan–Meier analyses and log rank tests (Mantel Cox). Correlations were quantified using the eta coefficient test and interpreted according to the conventional correlation coefficient scale [45].

## 3. Results

### 3.1. Survival and Irritation Analysis

**Figure 1:**
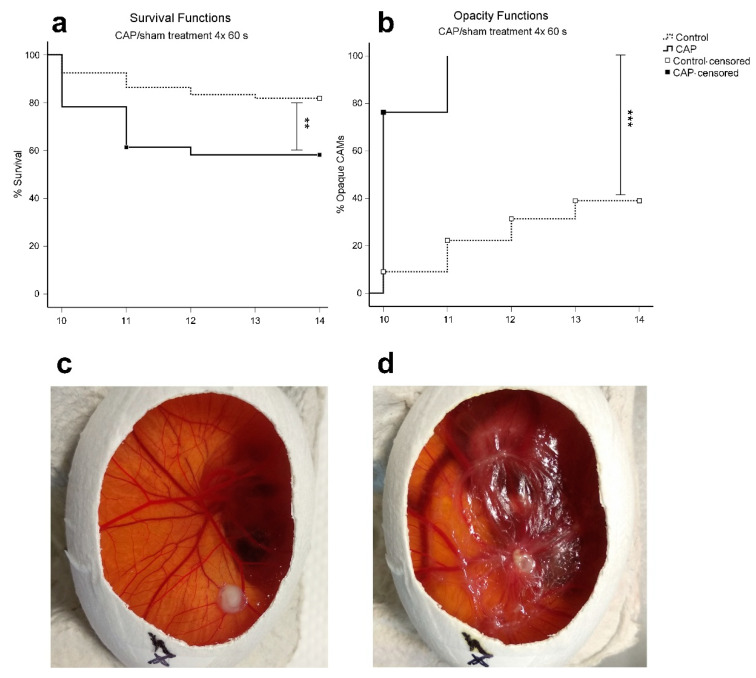
Kaplan–Meier curves of embryonal survival and CAM opacity after CAP treatment (*n* = 101) or sham treatment (*n* = 66). (**a**) The overall reduction of survival rate was highly significant (*p* = 0.001) after 4 × 60 s CAP treatment compared to sham treatment; (**b**) The rate of CAM opacity occurrence was significantly higher (*p* < 0.001) in CAP-treated eggs; (**c**) Representative images of an egg before CAP treatment on day 10 of incubation with clear CAM and a well-vascularized tumor (**d**) and past 4 × 60 s CAP treatment on day 14 of incubation with opaque and thickened CAM. ** *p* < 0.01, *** *p* < 0.001, in the log-rank test (Mantel–Cox).

To assess the hazards of CAP exposure on the CAM and the chicken embryo, survival of eggs and opacity of the tumor-bearing CAM treated with 60 s of CAP for four consecutive days (*n* = 101) was compared to sham treatment (*n* = 66). For the investigated time frame, a highly significant decrease (*p* = 0.001) in survival rate of CAP treated eggs was observed. CAP treated eggs demonstrated a survival rate of 58.4% on day 14 of incubation. In comparison, sham treated eggs showed a survival rate of 81.8% at the homologue observational time point (Figure 1a). The first two CAP treatments on days 10 and 11 of incubation led to a higher mortality (~20%) compared to the following treatments on days 12 and 13 of incubation (~2.5%). With respect to the opacity and thickening of CAM tissue, occurrence in CAP-treated eggs compared to sham-treated eggs was significantly increased (*p* < 0.001). After the first CAP treatment, opaque CAM tissue was observed in 76.2% (77/101) of all eggs, and after two CAP treatments, CAM tissue alterations were visible in the entire collective of treated eggs. In sham-treated eggs, the incidence of opaque CAM tissue was detected in 9.6% of all eggs per day (Figure 1b).

### 3.2. Immunohistochemical Analysis

#### 3.2.1. Vessel Density

**Figure 2:**
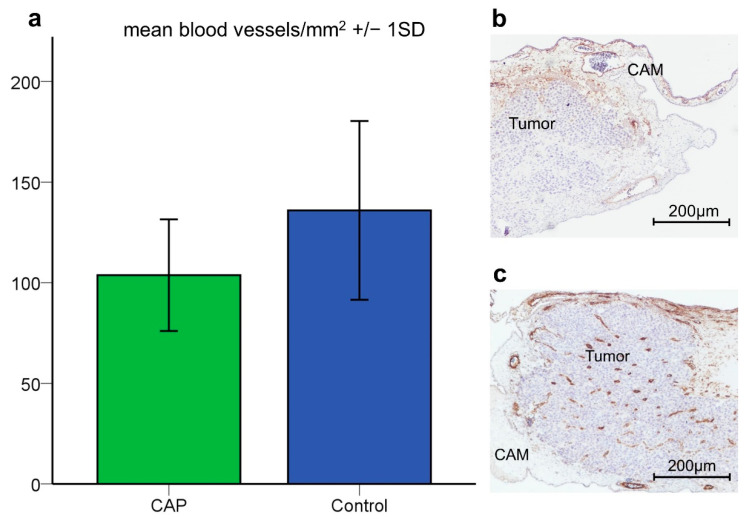
Vessel density in the tumor tissue. (**a**) The mean number of blood vessels per mm^2^ was significantly lower in tumors treated with 4 × 60 s CAP compared to sham-treated specimens. (**b**) Representative image of α-SMA-stained slide of low vascularized tumor tissue (CAP), scale bar length 200 µm. (**c**) Representative image of α-SMA stained slide of well-vascularized tumor tissue, scale bar length 200 µm (Control). *p* < 0.05.

The effects of CAP on tumor angiogenesis were analyzed in α-SMA-stained tumor slides after 4 × 60 s CAP (*n* = 44) or sham treatment (*n* = 51). Tumors treated with 4 × 60 s CAP demonstrated a significantly lower mean vessel density compared to tumors with sham treatment in the student’s *t*-test (*p* = 0.037) (CAP: Mean = 103.76/mm^2^, 27.75 (SD); sham: Mean = 135.93/mm^2^, 44.42 (SD)) (Figure 2a).

#### 3.2.2. Apoptosis

The effects of CAP on tumor cell viability in solid tumors were analyzed in cleaved caspase-3-stained tumor slides after 4 × 60 s CAP (*n* = 44) or sham treatment (*n* = 51). For the investigated tumor tissue, an overall significantly increased rate of apoptotic cells in CAP-treated tumors was observed with a Mann–Whitney U test (*p* = 0.005).

**Figure 3:**
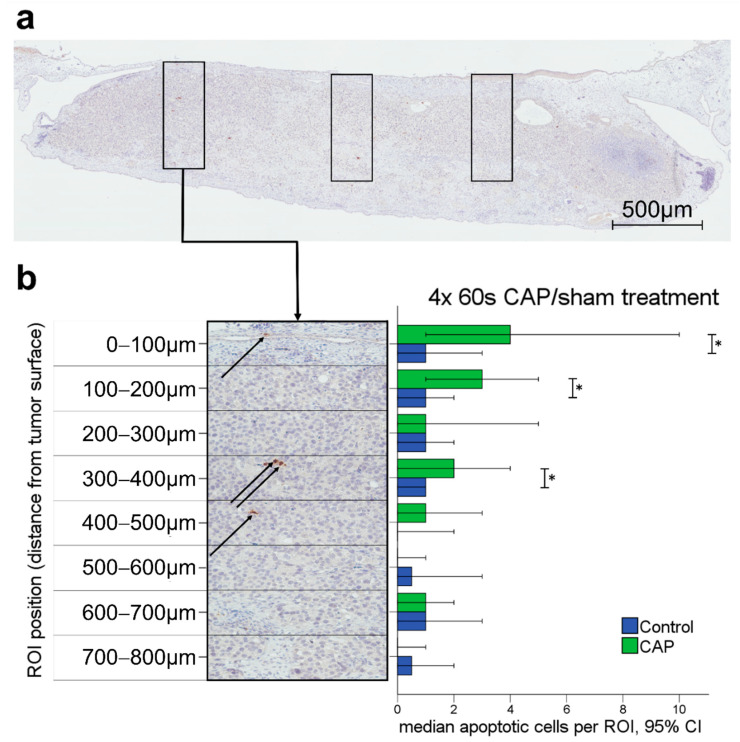
Apoptotic cell rates were analyzed in cleaved caspase-3 stained tumor slides. (**a**) Solid tumor in the CAM with three columns of ROI, scale bar length 500 µm; (**b**) Column of eight ROI with 400 × 100 µm each. Arrows indicate apoptotic cells. Median apoptotic cells by ROI position (distance from tumor surface). Significantly higher rates of apoptotic cells were observed in regions distanced less than 200 µm and between 300–400 µm from the tumor surface. * *p* < 0.05.

An increased apoptotic cell rate could mainly be observed in ROI located near the tumor surface (Figure 3b). More specifically, significantly higher rates of apoptotic cells in CAP-treated tumors were found up to a depth of 400 µm from the tumor surface, as displayed in Figure 3 (0–100 µm *p* = 0.012, 100–200 µm *p* = 0.048, 300–400 µm *p* = 0.027). The sample sizes of the analyzed ROI for each depth of tumor tissue are listed in Table S1. The depth dependency of CAP-associated effects was also confirmed using Kruskal–Wallis testing comparing apoptotic cell rates in depth-specific ROI within each treatment group (Supplementary Material S2).

### 3.3. VEGF Quantification

**Figure 4:**
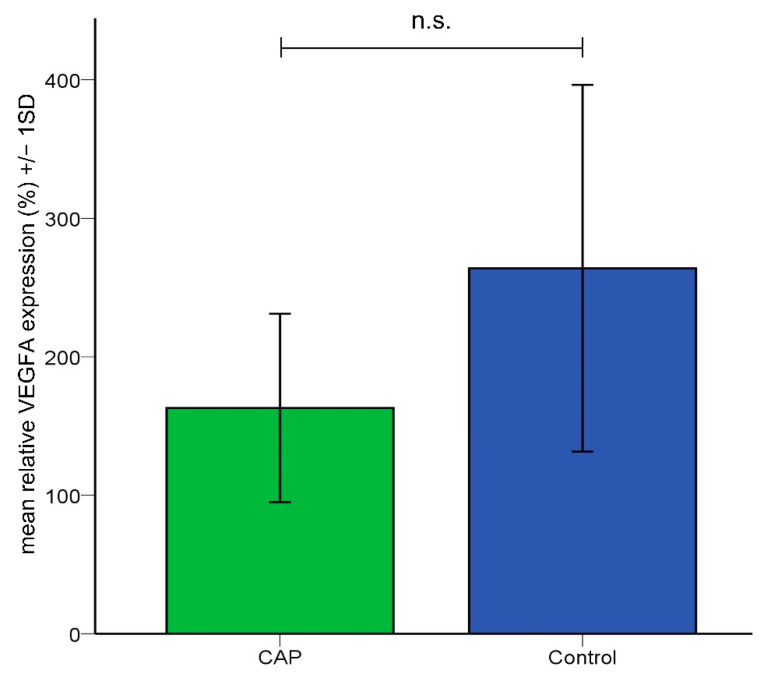
Mean relative VEGFA gene expression in the CAM tissue. Lower relative expression of VEGFA in CAM tissue treated with CAP was not significant compared to sham-treated tissue.

As the immunohistochemical analysis revealed a significant reduction of vessel density after CAP treatment in the tumor tissue, VEGF dependency was evaluated by quantification of VEGF mRNA using qRT-PCR. VEGF expression in the CAM tissue was not significantly reduced after CAP treatment in the quantitative RT-PCR (Figure 4). We compared native CAMs without tumors after 4 × 60 s CAP (*n* = 3) or sham treatment (*n* = 6). Mean relative VEGF gene expression was lower in CAP-treated CAM tissue (Mean = 163.03%) compared to sham-treated CAM tissue (Mean = 263.93%), but differences between groups did not reach the level of statistical significance (student’s *t*-test, *p* = 0.265) suggesting no or low effects of CAP on VEGF gene expression.

### 3.4. Ultrasonography

**Figure 5:**
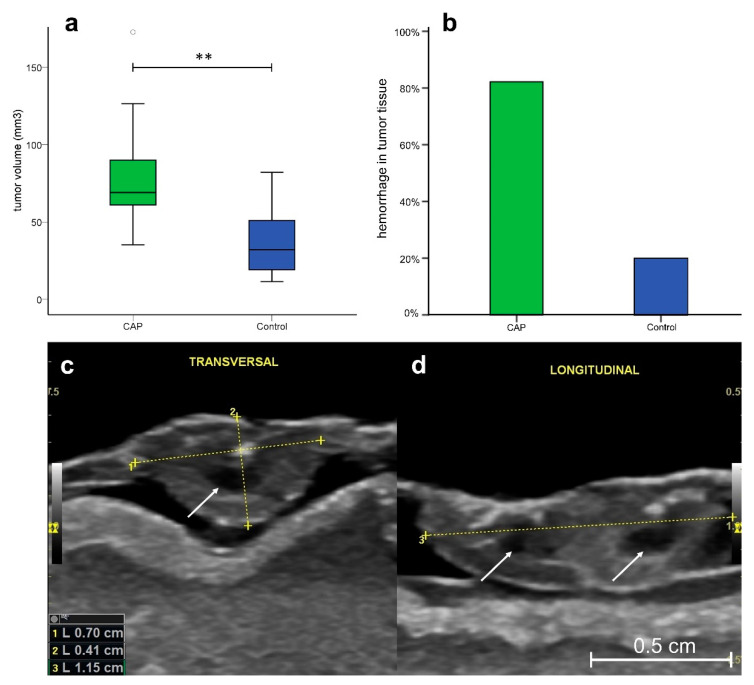
Ultrasonographically assessed tumor volume and hemorrhages. (**a**) Boxplot of tumor volume in specimens after CAP or sham treatment (CAP: *n* = 17, Median = 69.1, IQR = 31.9; sham: *n* = 15, Median = 32.1, IQR = 33.9). CAP-treated tumors demonstrated significantly increased tumor volume compared to untreated tumors (*p* < 0.01); (**b**) Intratumoral hemorrhage was observed in 82.4% of CAP-treated tumors and 20% of sham-treated tumors; (**c**) Ultrasonographic image of the inoculated tumor in CAM in the transversal axis (**d**) and the longitudinal axis. Scale bar length 500 µm. Arrows indicate intratumoral hemorrhage. ** *p* < 0.01.

Since intratumoral vessel density was reduced in the immunohistochemical analysis, supply with oxygen and nutrients might impair tumor growth. However, in vivo examination of tumors using ultrasonography demonstrated higher median tumor volumes in specimens treated with CAP (Median = 69.06 mm^3^ [CI 35.3–172.8]) compared to sham-treated tumors (Median = 32.07 mm^3^ [CI 11.5–82.1]) (*p* = 0.001) (Figure 5a).

Hemorrhages in the tumor tissue were detected in 82.4% (14/17) of tumors treated with CAP, while only 20% (3/15) of sham-treated tumors demonstrated intratumoral hemorrhage (Figure 5b). Regardless of prior treatment modality, subgroup analysis of tumor volume presented a significant correlation (η = 0.503, *p* = 0.03) between hemorrhage and tumor size in the eta coefficient test, indicating a hemorrhage-associated increase in tumor size.

### 3.5. Intravital Microscopy

**Figure 6:**
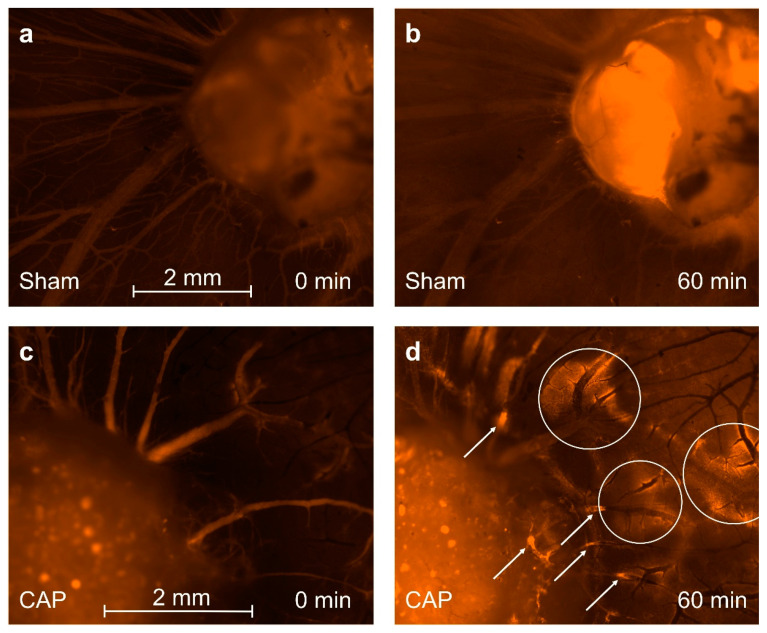
Intravital fluorescence imaging of tumor and surrounding blood vessels. Exemplary image of tumor (**a**) before and (**b**) past 60 min of 60 s sham treatment. A general increase of fluorescence could be observed. Intensification of fluorescence was mainly focused on tumor tissue and the CAM, distancing the tumor approximately 50 µm. Blood vessels surrounding the tumor appeared to be intact. Thrombosis or obliteration of blood vessels did not occur. Tumor (**c**) before and (**d**) past 60 min of 60 s CAP exposure. Increased extravasation of 70 kDa TRITC-dextran could be observed in tumor-associated CAM tissue (white circles). Vascular obliterations and thrombi occurred in several blood vessels (white arrows). Scale bar length 2 mm.

To further investigate the influence of CAP on vascular permeability and subsequent intratumoral hemorrhage, we evaluated vascular permeability by intravital microscopy in an explorative setting. Comparing repetitive images and video sequences of intravital fluorescence microscopy of tumor-associated blood vessels demonstrated an increased extravasation of TRITC-dextran (70 kDa) in addition to multilocular vessel occlusions after CAP treatment (Figure 6d). In contrast, the sham procedure did not result in vascular occlusion. Extravasation of TRITC-dextran could only be observed in the tumor adjacent vessels (Figure 6b). Fluorescence intensity of tumors successively increased over time regardless of the treatment applied. After CAP treatment, the onset of multilocular extravasation was detectable as early as 5 min after exposure. In comparison, only minimal extravasation could be observed after sham treatment and was detected at later time points (~20 min). These observations suggest a CAP-induced increase of vascular permeability in the tumor-associated blood vessels. In the analyzed video sequences, segmental blood flow indicated an intense decrease of blood flow immediately after CAP treatment. Whilst some vessels obstructed over time (~1 h) others demonstrated reconciliation after the initial decrease in flow (Figure S2).

## 4. Discussion

Antitumoral effects of CAP treatment on malignant cells have been elucidated by various working groups [4], however the influence of CAP on the tumor vasculature has only rarely been investigated.

In this study, significant changes in the vascular network within and surrounding the tumor were observed after CAP treatment of solid tumors. Beyond a reduced intratumoral vessel density, an increased vascular permeability, reduced blood flow and hemorrhages emphasizea profound effect of CAP on tumor vessels. Additionally, CAP resulted in an increased apoptosis of tumor cells at the tumor surface, indicating the direct hazardous effects of CAP on tumor cells.

Contrary to preliminary studies proclaiming CAP treatment was considered non-genotoxic and safe for clinical use, severe adverse effects of CAP treatment were observed when using the CAM assay as an experimental model. These effects were characterized by a significant reduction of chicken embryo viability (Figure 1a). Furthermore, CAP treatment resulted in the occurrence of irritations of the CAM tissue in the form of thickening and increased opacity (Figure 1b). Similar effects on chicken embryo viability and irritation of the CAM tissue by CAP have also been reported by others, suggesting possible hazards of CAP therapy [46,47]. However, when using the chicken embryo as a model organism, comparatively large areas of the embryonic vascular network are exposed to CAP treatment. The reduced viability after CAP treatment may be attributable to CAP-derived RONS. Though RONS can be detoxified by antioxidants present in the chicken embryo [48,49], the oxidative stress initiated by high CAP doses on such a large percentage of the CAM surface may overpower the antioxidant threshold of the embryo and eventually lead to major damage or death.

Tumor angiogenesis is a pivotal component tumor growth and nutritional supply [50]. Hence, the investigation of CAP-induced changes of tumor vasculature and the vascular network surrounding the tumor is of great importance. As displayed in Figure 2, tumors treated with CAP demonstrated a significantly lower mean vessel density compared to sham-treated tumors. These alterations seem to be attributed to direct effects like the coagulation of blood vessels [51,52,53] rather than to indirect effects such as reduced VEGF levels. The latter was indicated by a lack of significant differences in VEGF expression after CAP treatment (Figure 4) for. Contrary to these findings, reduced VEGF levels have been previously described in two malignant cell lines after CAP treatment [29,30].

By a reduction of tumor vascularization, which subsequently limits tumor progression by restricting the supply with oxygen and nutrients, CAP treatment may be utilized to facilitate antitumoral effects.

In accordance with our findings, Haralambiev et al. recently published data on the inhibition of endothelial cell migration and tube forming after CAP treatment, using an in vitro model [26]. Contrary to these findings, promotion of angiogenesis and microcirculation by CAP treatment of chronic wounds has been described for non-tumor vessels [54,55,56]. Whether CAP treatment may have a selective destructive effect on tumor vessels has yet to be determined. However, fragility and altered microvessel architecture of tumor vessels [57,58] presumably make them more sensitive to CAP-associated damage.

The induction of apoptotic cell death by CAP, as demonstrated in our experiments, has also been described by other working groups for malignant cell lines in vitro and in vivo [3,9]. CAP is known to mediate the induction of the intrinsic apoptosis pathway by disruption of the mitochondrial membrane, resulting in caspase activation, as observed in our experiments [59,60,61]. Longer exposure to CAP, however, has been proven to also induce caspase-independent cell death [62,63] and further antiproliferative effects like G1 phase cell cycle arrest [64]. Hence, the extent of tissue affection and apoptosis may be underestimated in our experiments, especially in areas of extensive CAP exposure. In the solid tumors, apoptosis occurred mainly in tissue areas close to the tumor surface as the reactive species form in the surrounding liquid and diffuse into the tumor tissue [11,34,65]. The effective depth of tissue penetration of CAP and its reactive components remains a subject of controversial discussion, though being rather essential information with respect to the treatment of solid tumors. Using the CAM assay as an experimental platform for solid tumors, Partecke et al. described the effects of CAP on tumor tissue to be limited to the upper three to five cell layers. They calculated a depth of tissue penetration to be at a maximum of 60 µm from the tumor surface [34]. Using marker-independent Raman microspectroscopy, more recent studies described CAP-dependent effects on DNA and lipids to a depth reaching the basal cell layer of epithelium in human tissue samples from the cervix uteri, suggesting a functional tissue penetration depth of roughly 270 μm [66,67]. The evaluated tissue samples in our experiments demonstrated increased apoptotic cell death in the tumor tissue up to a depth of 400 µm from the tumor surface (Figure 3b). Discrepancies may be influenced by differences in the CAP device and treatment times [68] and the fact that specific mechanisms related to CAP-associated tumor cell apoptosis are still not fully understood. Yet, in living tissue attached to a vascular network, apoptotic cell death in deeper tissue compartments could very well be caused by a combination of direct effects of CAP on tumor cells and indirect effects facilitated by damage of the supporting vascular network and an altered tumor angiogenesis.

In rodent models, other working groups reported a reduction of tumor volume after CAP treatment [20,22,69,70]. In discordance with these findings, we measured an increased volume of tumors treated with CAP with ultrasonographic imaging in comparison to sham-treated specimens (Figure 5a).

Ultrasonography has recently been demonstrated to be suitable for the measurement of tumor size in the CAM assay [35]. Since volume and intratumoral hemorrhage displayed a positive correlation in the eta coefficient test (η = 0.503, *p* = 0.03) this increase in tumor size is likely to be attributed to intratumoral hemorrhage rather than actual growth of the tumor after treatment. As demonstrated in Figure 5b, intratumoral hemorrhage was detectable in 82.4% of tumors treated with CAP. In comparison, only 20% of sham-treated tumors demonstrated hemorrhage within the tumor stroma.

The assumed vascular damage resembled by intratumoral hemorrhage is further supported by our observation of an increased TRITC-dextran extravasation after CAP exposure. Following CAP treatment, tumor-associated blood vessels of the CAM demonstrated an extensive extravasation of TRITC-dextran (70 kDa). Although a minimal extravasation was detectable after sham treatment in tumor-adjacent vessels, the extent of extravasation was extensively higher after CAP treatment, as demonstrated in Figure 6. Extravasation of TRITC-dextran (70 kDa) started approximately 5 min after treatment and perivascular accumulation of the fluorophore consecutively increased over time. CAP could conceivably damage tumor blood vessels and thereby cause altered permeability by damaging endothelial cell integrity [26]. In addition to damage of endothelial cells, changes in membrane permeability and function were observed in several cancer cell lines [71,72].

These effects are likely to be facilitated by the ability of CAP to induce pore formation in the cell membrane [73]. Furthermore, growth factors like VEGF [74,75] and pro-inflammatory factors are known to increase vascular permeability. As VEGF levels did not demonstrate significant differences in our experiments and other authors have reported decreased VEGF levels after CAP treatment [29,30], a VEGF-associated mediation seems unlikely. However, CAP is well known to induce multiple pro-inflammatory factors like interleukins, interferons, and tumor necrosis factors [76,77,78], creating an inflammatory milieu [76,79,80,81], which mediates an increase in vascular permeability and changes in blood flow [82,83,84,85,86]. Apart from these chemokines, nitric oxide (NO) is known to have a relevant influence on vascular permeability [87,88]. The levels of NO emitted by the used CAP device (miniFlatPlaSter^®^) are comparatively low [10,41]. Therefore, a mediation of the increase in vascular permeability by NO emitted from the CAP device seems unlikely. However, pro-inflammatory cytokines lead to the expression of the inducible NO synthase in inflammatory cells. Hence, higher levels of NO will likely be synthesized due to the CAP-associated induction of an inflammatory state in the tissue [89,90,91]. Whether the increase in vascular permeability is mainly driven by the electric potential induced by charge accumulation on the membrane or combined effects of chemical factors and the electric field has yet to be determined [92,93,94].

A strong initial decrease in flow was detectable immediately after CAP treatment. While some vessels resumed to a normal velocity of blood flow, others completely occluded over time, indicating an influence of CAP on microcirculation (Figure S2). Furthermore, multilocular vessel occlusions were visible one hour after treatment (Figure 6d). CAP has been demonstrated to influence blood coagulation in vitro and in vivo, which has been attributed to an enhanced fibrinogen aggregation [51,95]. More specifically, CAP-derived hydrogen peroxide may initiate haem-assisted crosslinking between fibrinogen molecules, thereby resulting in the formation of a fibrin network and ultimately blood coagulation [96]. By limiting the supply with oxygen and nutrients, vascular damage induced by CAP exposure may ultimately cause the observed increased intratumoral apoptosis.

The influence of CAP exposure on blood flow and vascular permeability of tumor-associated vessels has not been previously described. Yet, synergistic effects of nanoparticulate drugs or chemotherapeutics with CAP treatment in vitro and in vivo have been reported by other working groups [97,98,99]. These findings were mostly attributed to synergistic effects facilitated by the elevated levels of RONS leading to DNA damage and apoptosis [100]. However, in our opinion, a CAP-induced increase in vascular permeability could result in an increased accumulation of therapeutic drugs in solid tumors, which may also facilitate synergistic treatment effects of CAP and nanoparticles, chemotherapeutics or other systemic treatment options. It has to be considered that an increased vascular permeability could have detrimental effects for proper drug delivery, as an increase in tumor interstitial fluid pressure may limit the influx of the medication into the tumor tissue [74,101]. Yet, as the tightness of the tumor endothelium influences the efficacy of therapeutic drug delivery [74,102], simultaneous application of CAP and the therapeutic agent may allow a concerted manipulation of vascular permeability, increasing the efficiency of drug delivery by promoting infiltration of chemotherapeutics and nanoparticles into the tumor tissue.

Though some publications suggest a positive influence of CAP on tumor metastasis by regulating epithelial-mesenchymal transition [103] and down-regulation of pro-metastatic pathways [104,105,106], in vivo data is scarce and a negative influence of CAP-associated increase in vascular permeability cannot be dismissed.

Hence, considering the very mild local side effects of CAP treatment in clinical practice [107], further experiments should evaluate possible synergistic effects in addition to potential hazards of CAP combined with systemic treatment options. Localized, topical application of CAP may allow a targeted therapy focusing on the treated tumor area while sparing the surrounding tissue. Thus, effects of CAP treatment could substantially increase the efficacy of systemic therapeutics.

## 5. Conclusions

CAP has been demonstrated to reduce vascular density in solid tumors while increasing intratumoral apoptosis. CAP further increased the vascular permeability and attenuated the microcirculation by causing vessel occlusions in the tumor-associated vasculature. These effects further promote CAP as a promising augmentative treatment modality to systemic treatment approaches of solid tumors.

Although these findings were obtained in an explorative setup, the data obtained point out the potential of CAP as a promising and yet underrated therapeutic modality for addressing tumor vasculature in cancer therapy.

Further studies are urgently needed to evaluate and standardize plasma therapy in clinical practice. Therefore, the influence of vascular damage resulting in synergistic effects of CAP treatment with established and novel cancer therapies should be further investigated.

## Figures and Tables

**Table 1:** Primers used for quantitative real-time polymerase chain reaction.

Gene	RefSeq ID	Primer Name	Sequence
VEGFA	NM_205042.2	cVEGF3-s	AGAAAGGCCGGTACAAACCA
		cVEGF3-as	GCAAGTGCGCTCGTTTAACT
Beta-actin gene	ENSGALT00000015673	cACTB-s	ACCCCAAAGCCAACAGA
		cACTB-as	CCAGAGTCCATCACAATACC
HPRT1	AJ132697	cHPRT1-s cHPRT1-as	GCACTATGACTCTACCGACTATTG CAGTTCTGGGTTGATGAGGTT

## Data Availability

The data presented in this study are available on reasonable request from the corresponding author.

## Supplementary Materials

The following supporting information can be downloaded at: https://www.mdpi.com/article/10.3390/cancers14102432/s1, Figure S1: Experimental Setup; Supplementary Material S2: Depth dependency of apoptotic cells; Table S1: ROI sample sizes in varying depths of cleaved caspase-3 stained tumor slides; Figure S2: Segmental blood flow.
