# Supplementary material for: Cold Atmospheric Plasma Reduces Vessel Density and Increases Vascular Permeability and Apoptotic Cell Death in Solid Tumors
Source: Cancers (Basel). 2022 May 14;14(10):2432. doi: 10.3390/cancers14102432 (PMC9139209; doi:10.3390/cancers14102432)
Supplement: Supplementary file 1 [file cancers-14-02432-s001.zip › cancers-1703959-supplementary.pdf]

cancers

MDPI

Supplementary Material

Experimental Setup

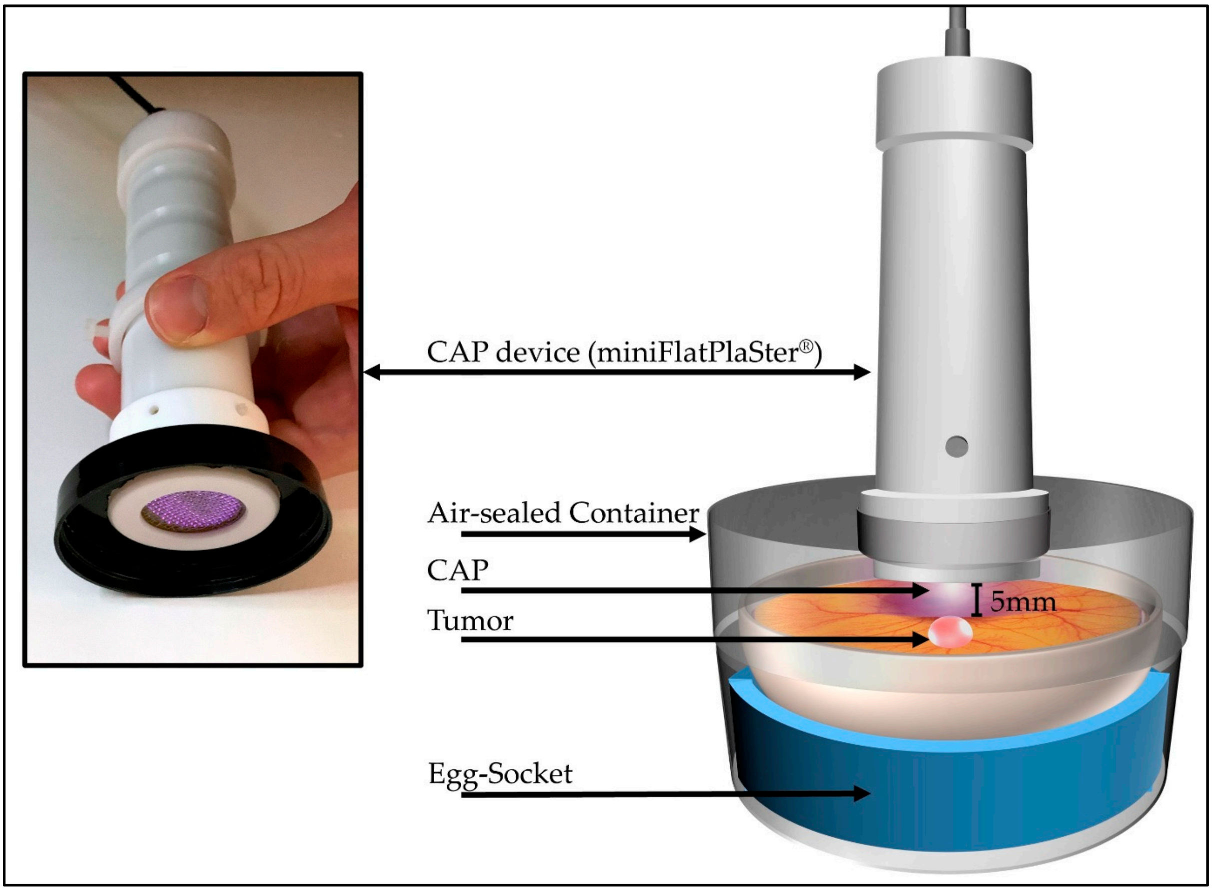

Figure S1. Tumor-bearing eggs were placed in a sealed container with the CAP device incorporated in the lid. This avoided air circulation and ensured the CAP device was located at an average distance of 5 mm from the CAM bearing the tumor.

Supplementary Material S2

Depth dependency of apoptotic cells

Depth dependency of CAP effects was confirmed using Kruskal-Wallis testing comparing apoptotic cell rates in depth-specific ROI within each treatment group. To evaluate the effective tissue penetration of CAP, Kruskal-Wallis testing comparing apoptotic cell rates in varying tissue depths was performed for both groups separately. CAP treated specimens showed to be highly significant ($p < 0.001$), while sham treated tumors did not show significant results ($p = 0.717$). For CAP specimens, Dunn-Bonferroni's post-hoc-tests with Bonferroni correction showed significant higher apoptotic cell rates in ROI within the upper 200 μm to ROI between 500–600 μm and 700–800 μm (0–100 μm*500–600 μm $p = 0.001$, 0–100 μm*700–800 μm $p = 0.004$, 100–200 μm*500–600 μm $p = 0.009$, 100–200 μm*700–800 μm $p = 0.021$).

Table S1. ROI sample sizes in varying depths of cleaved caspase-3 stained tumor slides.

distance from tumor surface	*n* (CAP)	*n* (Control)
0–100 μm	44	51
100–200 μm	43	50
200–300 μm	42	46
300–400 μm	34	38
400–500 μm	30	27
500–600 μm	27	14
600–700 μm	20	11
700–800 μm	17	8

Segmental blood flow

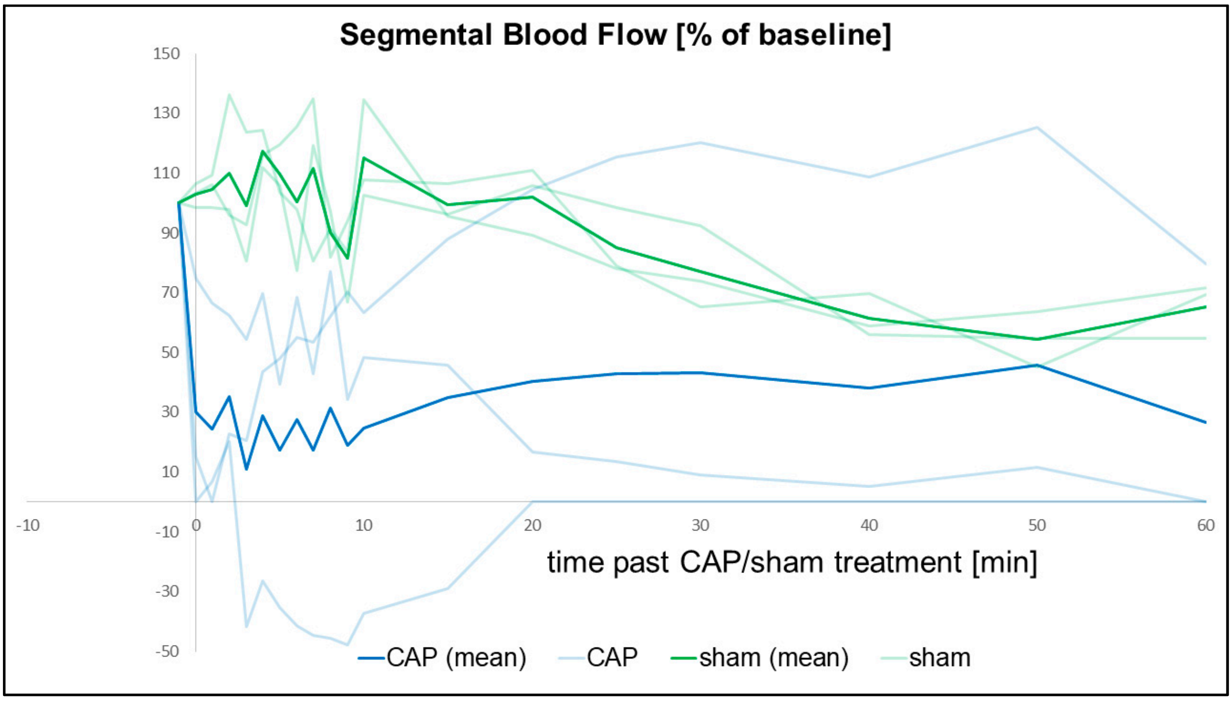

Figure S2. Changes of segmental blood flow in tumor associated blood vessels. Mean values in bold lines, individual values in thin lines (green: CAP, blue: sham). Immediate drop in segmental blood flow could be observed in all vessels after CAP exposure, with recovery to normal blood flow after 20 minutes in one blood vessel. Two vessels showed the occurrence of intravascular blood coagulation leading to a slower but irreversible decrease in blood flow and ultimate occlusion of the vessel. (CAP: n = 3, sham: n = 3)
